# Neurological soft signs are increased in major depressive disorder irrespective of treatment

**DOI:** 10.1007/s00702-023-02602-z

**Published:** 2023-02-20

**Authors:** Rasmus Schülke, Kyra Liepach, Anna Lena Brömstrup, Thorsten Folsche, Maximilian Deest, Stefan Bleich, Alexandra Neyazi, Helge Frieling, Hannah B. Maier

**Affiliations:** 1 Department of Psychiatry, Social Psychiatry and Psychotherapy, Hannover Medical School, Carl-Neuberg-Str. 1, 30625 Hannover, Germany; 2 Department of Psychiatry and Psychotherapy, Otto Von Guericke University, Magdeburg, Germany

**Keywords:** Major depressive disorder, Neurological soft signs, Electroconvulsive therapy, Antidepressant treatment

## Abstract

The significance of neurological soft signs (NSS) in major depressive disorder (MDD) remains unclear and the stability of NSS in relation to antidepressant treatment has never been investigated. We hypothesized that NSS are relatively stable trait markers of MDD. We thus predicted that patients show more NSS than healthy controls, irrespective of illness duration and antidepressant treatment. To test this hypothesis, NSS were assessed in chronically depressed, medicated MDD patients before (*n* = 23) and after (*n* = 18) a series of electroconvulsive therapy (ECT). In addition, NSS were assessed once in acutely depressed, unmedicated MDD patients (*n* = 16) and healthy controls (*n* = 20). We found that both chronically depressed, medicated MDD patients and acutely depressed, unmedicated MDD patients showed more NSS than healthy controls. The degree of NSS in both patient groups did not differ. Importantly, we found no change in NSS after on average eleven sessions of ECT. Thus, the manifestation of NSS in MDD seems to be independent of illness duration and pharmacological and electroconvulsive antidepressant treatment. From a clinical perspective, our findings corroborate the neurological safety of ECT.

**Supplementary Information:**

The online version contains supplementary material available at 10.1007/s00702-023-02602-z.

## Introduction

NSS are mild abnormalities of sensory integration, motor coordination, sequencing of complex motor acts and primitive reflexes that are found in a thorough neurological examination. Past research has focused on the significance of NSS in schizophrenia (Cox and Ludwig 1979a; Manschreck and Ames 1984; Heinrichs and Buchanan 1988; Bombin et al. 2005; Chan et al. 2010) and bipolar disorder (Goswami et al. 2006; Bora et al. 2018). However, increased NSS have also been found in obsessive compulsive disorder (Hollander et al. 2005; Karadag et al. 2011; Tumkaya et al. 2012), borderline personality disorder (De la Fuente et al. 2006), post-traumatic stress disorder (Gurvits et al. 1993, 2000, 2006; Belrose et al. 2020) and affective disorders (Cox and Ludwig 1979b; Gureje 1988; Cherian and Kuruvilla 1989). Some signs, such as deficits in stereognosis and rhythm tapping, have even been found more frequently in mood disorders than in schizophrenia (Boks et al. 2000). For late-onset MDD (first episode at ≥ 50 years, age at assessment > 60 years), a higher prevalence of NSS in patients compared to healthy controls has been demonstrated (Baldwin et al. 2005). Furthermore, symptoms of catatonia (Starkstein et al. 1996) and Parkinsonism (Starkstein et al. 2001) were found in 20% of MDD patients. In contrast to these findings, a study including patients with schizophrenia, bipolar disorder and unipolar depression did not find a NSS difference between healthy controls and moderately depressed patients (Zhao et al. 2013). Likewise, another recent study found no difference in total NSS scores between unipolar patients and controls (Sagheer et al. 2018). Overall, very few studies have investigated NSS in unipolar depression. The samples of depressed patients have often been insufficiently characterized, even with regard to symptom severity (Sagheer et al. 2018), included both unipolar and bipolar patients (Gureje 1988; Cherian and Kuruvilla 1989; Boks et al. 2000, 2004) and were not followed-up longitudinally. Therefore, the significance of NSS in MDD remains unclear. In schizophrenia, NSS are found over the entire course of the disorder and have been suggested to constitute an endophenotype (Bombin et al. 2005), even though this view has been questioned, given that NSS have been found to decrease with remission of psychopathological symptoms (Bachmann et al. 2014; Bachmann and Schröder 2018). Importantly, NSS have been found in first-episode psychosis (Dazzan and Murray 2002; Boks et al. 2004; Mouchet-Mages et al. 2011) and are present independent of antipsychotic treatment (Gupta et al. 1995; Arango et al. 2000; Dazzan and Murray 2002; Venkatasubramanian et al. 2003). Regarding MDD, NSS have to our knowledge never been studied longitudinally and in relation to ECT treatment before.

In line with the proposed role of NSS in schizophrenia, we hypothesized that neurological abnormalities in MDD are trait rather than state markers and should thus be independent of antidepressant treatment and illness duration. To test this hypothesis, we first compared the prevalence of NSS in (chronically) depressed medicated MDD patients, (acutely) depressed unmedicated MDD patients and healthy controls. We hypothesized that patients would manifest more NSS than controls but expected no difference in NSS between chronically depressed, medicated patients and acutely depressed, unmedicated patients. We then compared pre- and post-ECT NSS scores in the group of chronically depressed, medicated patients. Based on our hypothesis, we expected no change in NSS after electroconvulsive therapy.

## Materials and methods

### Participants

This study was conducted at the Department of Psychiatry, Social Psychiatry and Psychotherapy at the Hannover Medical School (Germany) and approved by the ethics committee of the Hannover Medical School (Ethikkommission der Medizinischen Hochschule Hannover, registration number: 2842–2015). The study was carried out in accordance with the latest version of the Declaration of Helsinki. All participants were in capacity to give consent and gave written informed consent before inclusion in the study. Chronically depressed, medicated MDD patients scheduled for ECT (ECT group, *n* = 23), acutely depressed, unmedicated MDD patients (UDC group, *n* = 16) and healthy controls (HC group, *n* = 20) were included in the study (see Table 1 for sample characteristics). Patients (UDC and ECT group) were screened for study inclusion after hospitalization. Patients with MDD, diagnosed according to the ICD-10 (International Statistical Classification of Diseases and Related Health Problems 10th Revision) criteria, i.e., ICD-10: F32.2/F32.3/F33.2/F33.3, were included in the study. Additional inclusion criteria were either scheduled ECT treatment (ECT group) or no current psychopharmacological medication (UDC group). Healthy controls were recruited via notices distributed in supermarkets and at the university. All healthy controls had a history free of mental or neurologic illness and alcohol or drug abuse. The ECT cohort was drawn from the prospective Northern German Electroconvulsive Therapy Outcome Registry (Norddeutsches Elektrokonvulsionstherapie Outcome Register [NEKTOR]). Education was assessed on a 4-level scale (0: no school leaving qualification, 1: certificate of secondary education, 2: general certificate of secondary education, 3: general qualification for university entrance or vocational diploma). To assess depression severity, the MADRS (Montgomery-Åsberg Depression Rating Scale) and BDI-II (Beck’s Depression Inventory) were used. All patients continued their regular pharmacological treatment during ECT. Of the 23 medicated ECT patients, 22 (95.7%) were on antidepressants, 7 (30.4%) on benzodiazepines, 19 (82.6%) on antipsychotics and 2 (7.4%) on Lithium (plasma concentration < 0.5 mmol/L). Of the patients prescribed antipsychotics, 15 were taking atypical antipsychotics, and 10 were taking typical antipsychotics. However, typical antipsychotics were only prescribed as on-demand medication, with most patients taking pipamperone (occasionally and in low doses). The majority of patients were taking multiple psychopharmacological drugs (1 drug: 3 patients (13.0%), 2 drugs: 8 patients (34.7%), 3 drugs: 4 patients (21.7%), 4 drugs: 5 patients (21.7%), 5 drugs: 1 patient (4.3%), 6 drugs: 2 patients (8.7%)).

**Table 1:** Sample characteristics. HC: healthy controls, UDC: unmedicated, acutely ill MDD patients, ECT: medicated, chronically ill MDD patients who underwent electroconvulsive therapy (assessed pre- and post-ECT)

Timepoint	Group	Variable		*n*	Min	Max	Median	Mean	SD
Sample Characteristics									
Pre	HC	Females/Males	12/8						
		Education	0/0/9/11						
		Age	20		23.0	66.00	39.00	43.55	14.63
		BMI	20		19.0	46.00	25.00	25.70	6.10
		BDI-II	20		0.0	5.00	2.00	2.05	1.70
		MADRS	20		0.0	2.00	0.00	0.10	0.45
		NSS	20		11.0	38.00	17.50	21.00	8.56
	UDC	Females/Males	11/5						
		Education	0/2/5/9						
		Age	16		20.0	62.00	49.50	45.62	14.42
		BMI	16		20.0	41.00	26.50	26.38	5.16
		Current episode (weeks)	12		2.0	156.00	8.50	30.00	48.48
		BDI-II	16		5.0	45.00	25.50	25.50	12.08
		MADRS	16		8.0	43.00	27.50	28.69	9.02
		NSS	16		15.0	71.00	33.50	34.69	15.68
	ECT	Females/Males	13/10						
		Education	0/2/8/11						
		Age	23		26.0	65.00	51.00	47.91	11.08
		BMI	22		20.7	43.03	28.53	29.03	5.78
		Current episode (weeks)	16		2.0	938.00	91.00	187.88	279.96
		BDI-II	23		16.0	48.00	38.00	36.91	8.99
		MADRS	23		12.0	43.00	29.00	28.30	8.34
		NSS	23		16.0	62.00	33.00	37.35	13.18
post		Females/Males	12/6						
		Education	0/0/8/8						
		Age	18		26.0	64.00	50.00	47.61	10.97
		ECT sessions	18		5.0	18.00	12.00	11.00	2.93
		BDI-II	16		0.0	43.00	22.50	23.44	15.47
		MADRS	18		1.0	43.00	20.00	18.22	10.95
		NSS	18		23.0	75.00	31.50	36.78	12.80

Education was assessed on a 4-level scale: no school leaving qualification/certificate of secondary education/general certificate of secondary education/general qualification for university entrance or vocational diploma. BMI: Body mass index. BDI-II: Beck’s Depression Inventory II. MADRS: Montgomery-Åsberg Depression Rating Scale. NSS: Neurological soft signs.

### Electroconvulsive therapy

ECT was applied according to standard clinical practice, with two or three sessions per week being applied over the course of four to five weeks. The brief-pulse Thymatron System IV (Somatics, Lake Bluff, Illinois, USA) was used for stimulation. Initial stimulation dose was determined using the age-based method. ECT treatment was performed with right unilateral electrode placement for all ECT patients, with the exception of one patient who was switched from right unilateral to bitemporal stimulation for the last two sessions. Methohexital or propofol were used for anesthesia, remifentanil was used for analgesia, and succinylcholine, mivacurium or rocuronium were used for muscle relaxation. During ECT, an electroencephalogram (EEG) with two channels was recorded to measure seizure duration. In case of insufficient duration, stimulation intensity was increased. On average, 11.00 ECT sessions (SD = 2.93) were conducted. A reduction of ≥ 50% in MADRS scores was defined as response and a MADRS score < 10 was considered as remission.

### Neurological soft signs

Neurological soft signs were assessed using the comprehensive scale developed by Gurvits et al. (Gurvits et al. 1993). This NSS scale comprises 50 items and has successfully been employed in several studies investigating NSS prevalence in chronic posttraumatic stress disorder (Gurvits et al. 1993, 2000, 2006), see Online Resource 1. It is an extended version of the Neurological Evaluation Scale (Buchanan and Heinrichs 1989) and was chosen for its comprehensiveness (50 items compared to 34 items of the Heidelberg Scale (Schröder et al. 1992) and 41 items of the Neurological Evaluation Scale (Buchanan and Heinrichs 1989)). The scale takes about 45–60 min to perform. The 50 signs examined focus on sensory integration, motor coordination and sequencing of complex motor acts. NSS were scored by trained raters on a scale from 0 (no error or unremarkable) to 3 (3 or more errors or very noticeable). Raters were not blind to diagnostic status. The total NSS score for each participant was calculated as the sum of scores for all 50 individual items, resulting in a scale ranging from 0 to 150 points.

### Study design

NSS were assessed in a group of medicated, chronically depressed patients before and after a full course of ECT. In addition, a group of unmedicated, acutely depressed controls and a group of matched healthy controls, both matched with regards to age, sex and education, were recruited and assessed once. In the ECT group, post-ECT NSS assessments were performed when patients were still hospitalized, usually a few days after the last ECT session.

### Missing data

Of the initial *n* = 23 ECT patients, five patients did not show up for follow-up assessment after their ECT series, the sample size for follow-up analyses was thus reduced to *n* = 18. Missing values regarding the clinical and socio-demographic data (BDI-II: 2, current episode duration: 11, education: 3, BMI: 1; see Table 1) were excluded from the respective analyses.

### Statistical analysis

All statistical analyses were performed in* R *(version 4.0.4). First, we compared overall NSS scores across healthy participants, unmedicated acutely depressed patients and chronically depressed patients scheduled for ECT. Cronbach’s alpha with bootstrapped confidence intervals was computed across all three groups as a measure of internal consistency of the applied NSS scale. Since the normality assumption was violated for all variables except age, as assessed by QQ plots and Shapiro–Wilk tests, non-parametric Kruskal–Wallis *H* tests and Mann–Whitney *U* tests were performed for comparisons of the three groups. Potential socio-demographic differences between the groups were tested for by ANOVA (age), Fisher's exact test (sex distribution), Kruskal–Wallis *H* tests (education, body mass index) and Mann–Whitney *U* test (episode duration). Kruskal–Wallis *H* tests were performed to compare total NSS, MADRS and BDI-II scores. These were followed-up with Bonferroni-corrected, pairwise Mann–Whitney *U* tests. We performed the two-sided versions of all tests. Based on our hypotheses, we were mainly interested in whether NSS scores differed between chronically-depressed ECT patients, acutely-depressed patients and healthy controls. After comparing total NSS scores between the groups, we performed Kruskal–Wallis *H* tests for all 50 specific signs. Here, the Benjamini–Hochberg method was applied to control the false discovery rate. Significant results were followed-up with two-sided, Bonferroni-corrected Mann–Whitney *U* tests. To further explore potential associations of NSS with depressive symptoms and age, Spearman's rank correlation coefficients between total NSS scores and MADRS scores, BDI scores and age were computed. To rule out potential confounding, we then calculated partial Spearman correlation coefficients between total NSS scores and MADRS/BDI scores while controlling for age. Exploratively, we also performed two-sided Mann–Whitney *U* tests to probe whether the amount of manifested NSS might differ between patients on antipsychotics and patients not on antipsychotics, as well as between patients on benzodiazepines and patients not on benzodiazepines. Next, we tested whether NSS scores changed over the course of electroconvulsive therapy in ECT patients. Two-sided Wilcoxon signed-rank tests were performed to compare total NSS scores of ECT patients pre- and post-ECT. Differences concerning the 50 individual signs were also assessed using two-sided Wilcoxon signed-rank tests. The Benjamini–Hochberg method was applied to control the false discovery rate. Exploratively, to test whether NSS increases post-ECT might be more common in elderly patients, we performed a t-test to probe whether age differed between patients who manifested more NSS post-ECT and patients who manifested less NSS post-ECT. For all Mann–Whitney *U* and Wilcoxon signed-rank tests the Wilcoxon effect size* r *was computed (small effect: 0.1–< 0.3, moderate effect: 0.3–< 0.5, large effect: ≥ 0.5).

## Results

There were no differences between healthy participants (*n* = 20), unmedicated acutely depressed patients (*n* = 16) and chronically depressed patients scheduled for ECT (*n* = 23) regarding age (ANOVA: F(2, 56) = 0.579, * p *= 0.564), sex distribution (Fisher's exact test: * p *= 0.749) education (Kruskal–Wallis *H* test: H(2) = 0.183, * p *= 0.913) and body mass index (H(2) = 4.94, * p *= 0.085), see Table 1. The current depressive episode lasted longer in the ECT compared to the UDC group (Mann–Whitney *U* test, *U* = 43, * p *= 0.015, *r* = 0.466). All three groups differed regarding MADRS scores (H(2) = 40.4, * p *< 0.001). Both ECT (*U* = 0, *p*_adj_ < 0.001, *r* = 0.894) and UDC patients (*U* = 0, *p*_adj_ < 0.001, *r* = 0.919) showed more depressive symptoms than healthy controls. There was no difference in MADRS scores between the ECT and UDC patient groups (*U* = 194, *p*_adj_ = 1, *r* = 0.044). All three groups also differed regarding BDI-II scores (H(2) = 42.7, * p *< 0.001). Both ECT (*U* = 0, *p*_adj_ < 0.001, *r* = 0.857) and UDC (*U* = 0.5, *p*_adj_ < 0.001, *r* = 0.850) patients reported more depressive symptoms than healthy controls. However, ECT patients also reported more symptoms than UDC patients (*U* = 82.5, *p*_adj_ = 0.012, *r* = 0.465). Cronbach’s alpha, computed across all three groups, indicated good internal consistency of the NSS scale (*α* = 0.88, 95% CI [0.82, 0.91]).

### Increased neurological soft signs in unmedicated, acutely ill and medicated, chronically ill patients

**Fig. 1:**
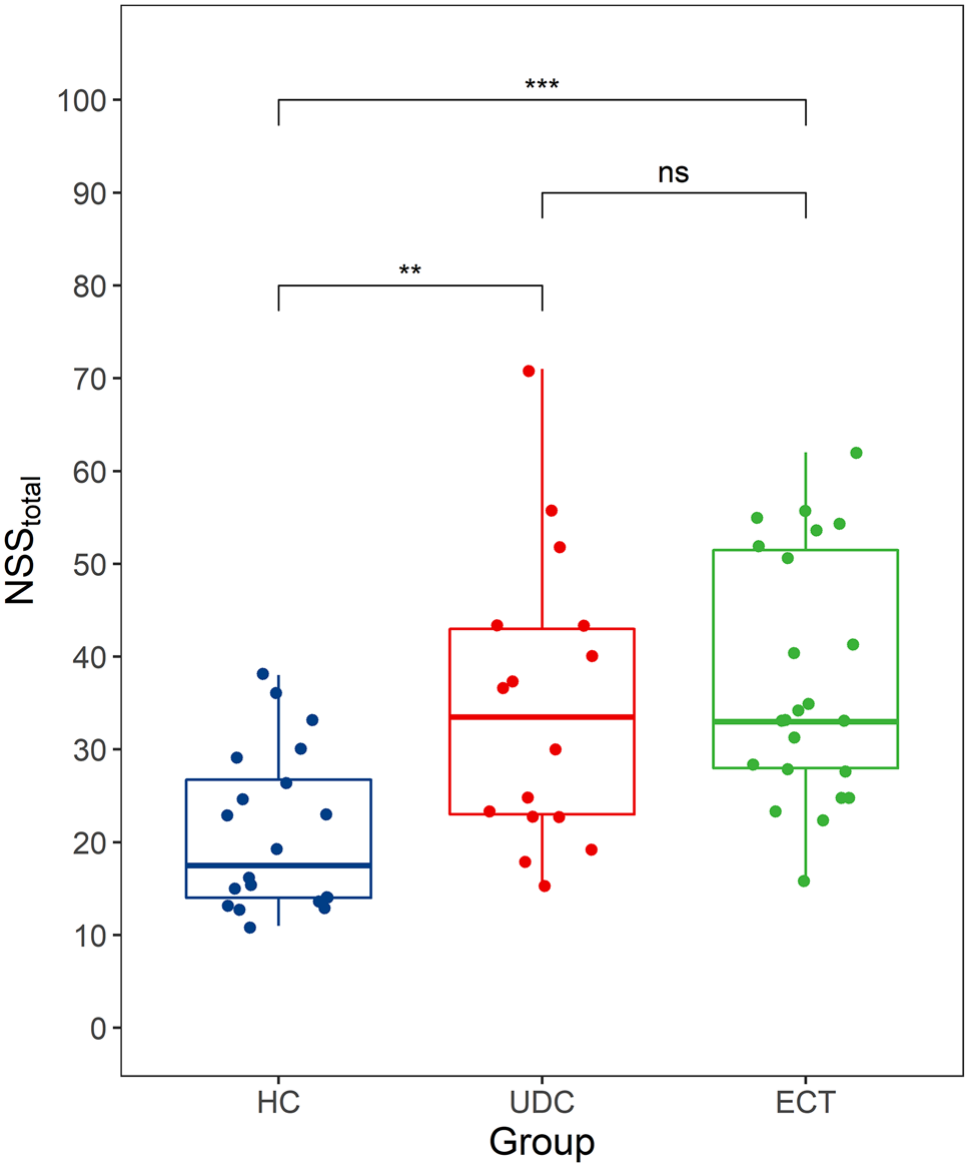
Neurological soft signs in patients and controls**.** Shown are total NSS scores for chronically ill, medicated patients scheduled for electroconvulsive therapy (ECT), acutely ill, unmedicated patients (UDC) and healthy controls (HC). Bonferroni-adjusted Mann–Whitney *U* tests were performed for pairwise comparisons. ***p_adj_ < .001, **p_adj_ < .01

**Fig. 2:**
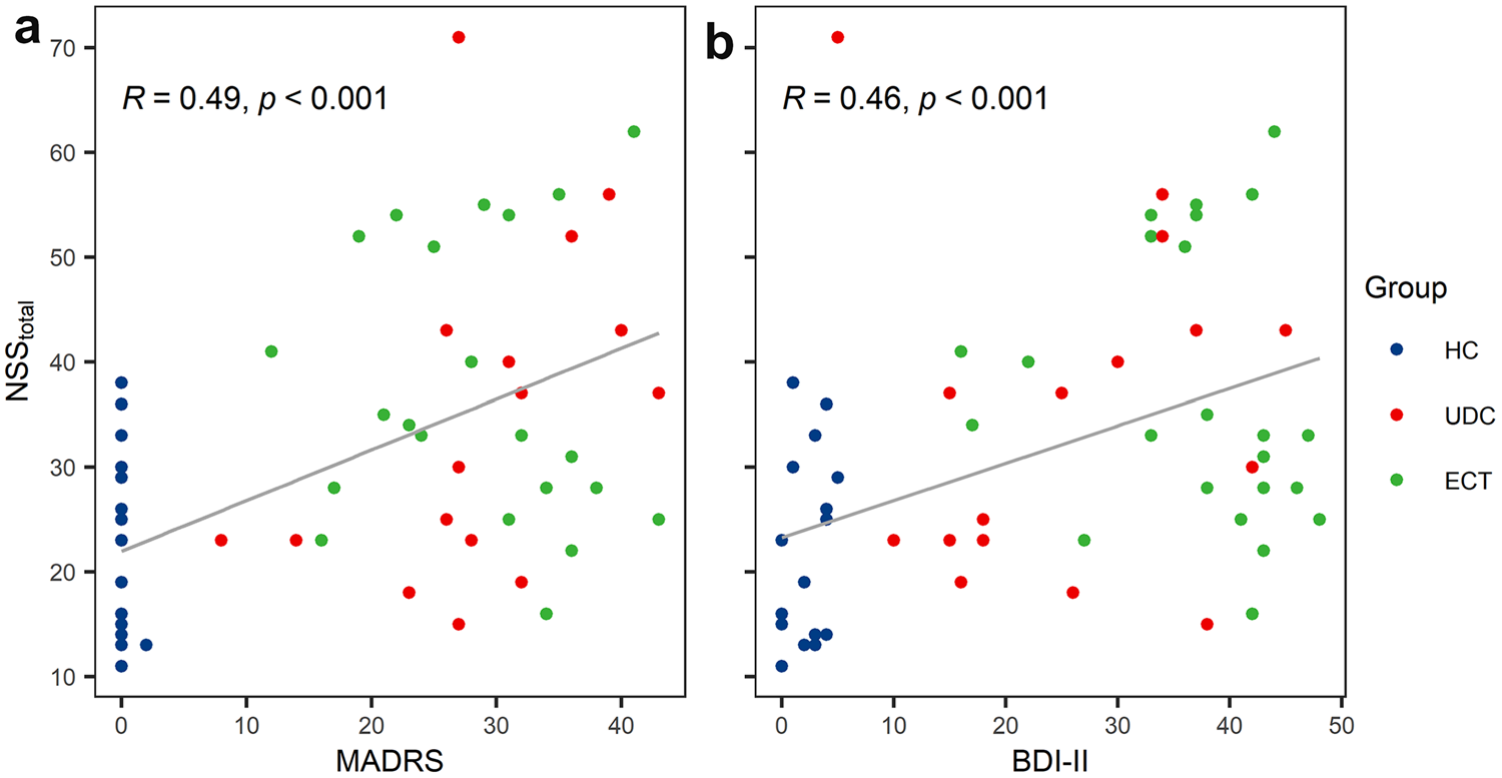
Correlation of depressive symptoms and neurological soft signs. Shown are Spearman's rank correlations of MADRS (**a**) and BDI-II (**b**) scores with total NSS scores for chronically ill, medicated patients scheduled for electroconvulsive therapy (ECT), acutely ill, unmedicated patients (UDC) and healthy controls (HC)

In line with our hypothesis about increased NSS in patients with MDD, we found a difference between the three groups regarding total NSS scores (Kruskal–Wallis *H* test: H(2) = 16.9, * p *< 0.001). Bonferroni-corrected, two-sided post-hoc Mann Whitney *U* tests indicated that both the ECT (*U* = 70, *p*_adj_ < 0.001, *r* = 0.595) and the UDC (*U* = 67.5, *p*_adj_ = 0.010, *r* = 0.492) group manifested more NSS than healthy controls, whereas there was no difference between the ECT and UDC groups (*U* = 158, *p*_adj_ = 1, *r* = 0.119; see Fig. 1). We also found a group difference – after FDR-correction—for 9 out of 50 individual NSS (see Online Resource 2). Post-hoc pairwise comparisons showed that UDC patients scored higher than controls on 4 signs, whereas ECT patients scored higher than controls on 8 signs. A difference between the two patient groups was only found for a single sign (graphesthesia L), due to a higher score in the ECT group. These results thus closely followed the comparison of overall NSS scores. Looking at the relationship between total NSS score and MADRS, BDI-II and age, across all subjects, we found moderate to large positive Spearman correlations of individual total NSS scores with all three variables (MADRS: *r* = 0.49, *p* < 0.001, see Fig. 2; BDI-II: *r* = 0.46, *p* < 0.001, Age: *r* = 0.43, *p* < 0.001). The Spearman correlation coefficients between total NSS and MADRS and BDI-II remained largely unchanged when controlling for age by performing partial correlations (MADRS: *r* = 0.48, *p* < 0.001; BDI-II: *r* = 0.48, *p* < 0.001). We did not find a difference in total NSS scores between patients taking antipsychotics (ap +) and patients not taking antipsychotics (ap-; Mdn_ap-_ = 38, Mdn_ap+_  = 33, n_ap-_ = 4, n_ap+_  = 19, *U* = 35.5, *p* = 0.871, *r* = 0.042) or between patients taking benzodiazepines (bd +) and patients not taking benzodiazepines (bd-; Mdn_bd-_ = 37.5, Mdn_bd+_  = 28, n_bd-_ = 16, n_bd+_  = 7, *U* = 79.5, *p* = 0.123, *r* = 0.328).

### No change in neurological soft signs after ECT

**Fig. 3:**
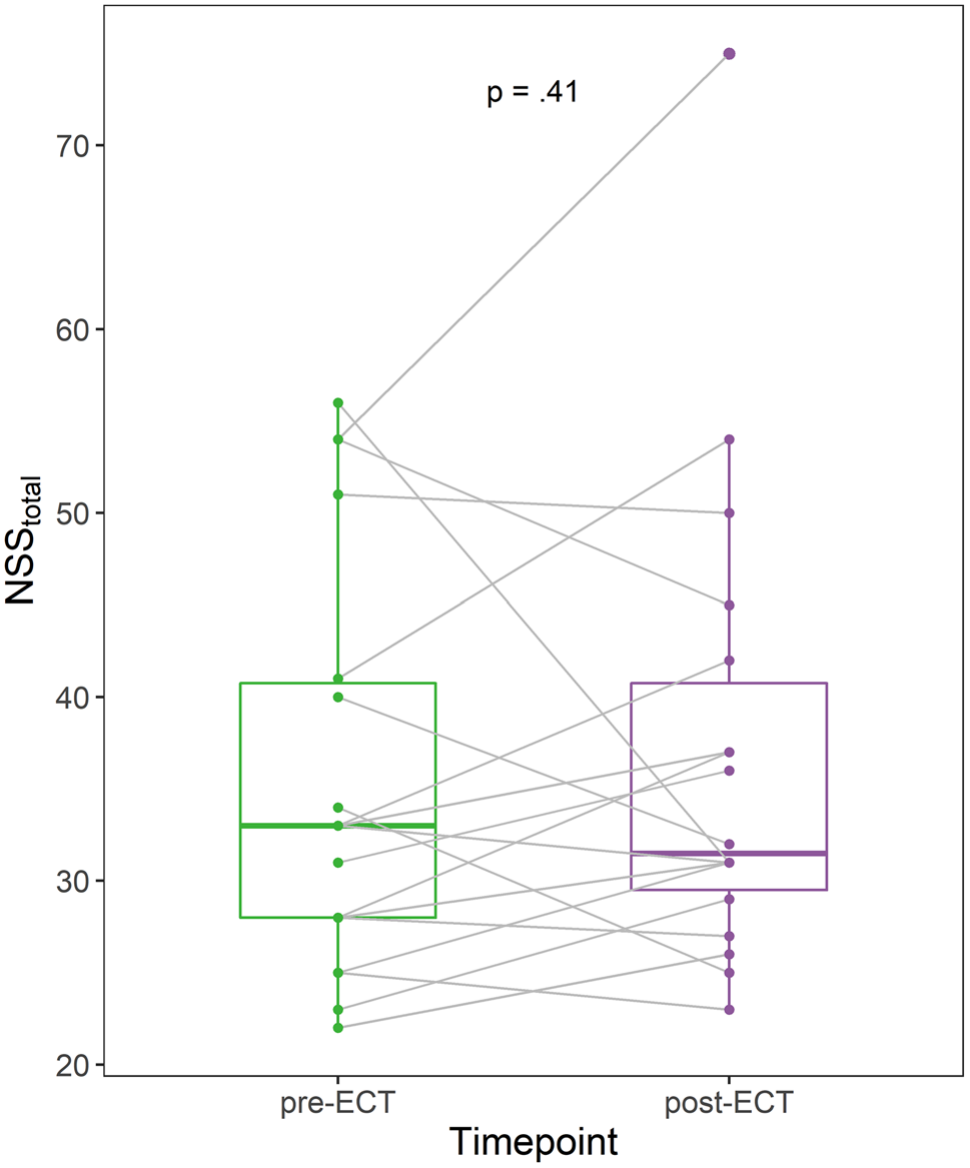
Neurological soft signs before and after electroconvulsive therapy. Shown are total NSS scores for the group of chronically ill, medicated patients before (pre-ECT) and after (post-ECT) electroconvulsive therapy. A Wilcoxon signed-rank test was performed for pre-post comparison

NSS scores after the full course of ECT (*M* = 11.00 sessions, SD = 2.93) were available for 18 patients. As expected, both MADRS (Wilcoxon signed-rank test, *V* = 138, * p *= 0.004, r = 0.694) and BDI-II (V = 113, * p *= 0.003, r = 0.770) scores decreased after ECT. Six patients (33%) responded to ECT and five patients (28%) remitted. Importantly, we found no difference between total NSS scores pre- and post-ECT (V = 66, * p *= 0.407, r = 0.200), despite considerable changes in individual patients (see Fig. 3). Likewise, concerning the 50 individual NSS signs, the two-sided Wilcoxon signed-rank tests (FDR-corrected) did not result in any pre-post-ECT differences. Even without FDR-correction, only the three comparisons for graphesthesia (L), graphesthesia (*R*) and foot tapping (R) became significant (graphesthesia R: *V* = 0, * p *= 0.002; graphesthesia L: V = 18, * p *= 0.027, foot tapping R: V = 40.5, * p *= 0.025), with higher post-ECT scores for graphesthesia and lower post-ECT scores for foot tapping. Exploratively, the age of patients showing less NSS post-ECT did not differ from the age of patients showing more NSS post-ECT (M_NSS↓_ = 46.6, SD = 12.2, M_NSS↑_ = 48.4, SD = 10.5, t(14.0) = -0.327, * p *= 0.749, *d* = -0.156).

## Discussion

In this study, we investigated the significance of neurological soft signs in MDD. We found that MDD patients showed more NSS than healthy controls. There was, however, no difference between acutely ill, unmedicated patients and chronically ill, medicated patients. Interestingly, NSS remained unchanged in the chronically ill patients after on average 11 sessions of ECT and substantial amelioration of depressive symptoms.

Even though NSS have been most thoroughly investigated in schizophrenia, increased NSS have also been reported in obsessive compulsive disorder (Hollander et al. 2005; Tumkaya et al. 2012), borderline personality disorder (De la Fuente et al. 2006), post-traumatic stress disorder (Gurvits et al. 1993, 2000, 2006) and MDD (Cox and Ludwig 1979b; Baldwin et al. 2005). NSS in unipolar depression have so far received little attention, despite the fact that psychomotor retardation is a core feature of depression (Sobin and Sackeim 1997) and part of DSM-V criteria for MDD diagnosis. While much research has focused on potential cognitive side effects of ECT (Semkovska and McLoughlin 2010), NSS had hitherto never been studied longitudinally in MDD patients receiving ECT. Given a prevalence of catatonia and symptoms of Parkinsonism in depressed patients of up to 20% (Starkstein et al. 1996, 2001), it is not surprising that we found marked impairment regarding fine motor skills, motor coordination and sequential movements. Interestingly, we found that NSS were present to a similar extent in acutely ill patients not yet on medication and chronically ill, medicated patients. Furthermore, NSS scores remained unchanged even after a full course of ECT. If NSS in depressed patients were a mere epiphenomenon of depressive episodes, ECT, the most effective antidepressant treatment available (UK ECT Review Group 2003), should have reduced NSS alongside depressive symptoms. Together, our results seem to indicate that NSS in MDD may be quite stable over the course of the disorder. This would parallel findings in schizophrenia, where NSS are already present in first-episode psychosis (Dazzan and Murray 2002; Boks et al. 2004; Mouchet-Mages et al. 2011), persist over the whole course of illness (Bombin et al. 2005; Bachmann and Schröder 2018) and are hardly affected by antipsychotic treatment (Arango et al. 2000; Bombin et al. 2005). However, it should also be noted that NSS deteriorate in the long term in patients with chronic schizophrenia (Herold et al. 2021) while improving in patients that show a remitting course of the disorder (Bachmann et al. 2014; Bachmann and Schröder 2018). It remains an open question to what extent NSS may also deteriorate over the long term in different subgroups of MDD patients. Supporting a trait-like nature of NSS in MDD, the manifestation of NSS during childhood predicts later manifestation of anxiety and depression (Shaffer et al. 1985). Furthermore, a recent study comprising 10,835 adolescents aged 9–11 years not only found that youths with motor abnormalities had an increased familial depression risk but were also more likely to develop depressive symptoms at 1-year follow-up (Damme et al. 2022). In our study, data on familial depression risk, early childhood adversity and early neurological abnormalities is missing, so more studies are needed to clarify the potential role of NSS as vulnerability markers of unipolar depression. Moreover, to what extent neurological abnormalities might actually be a shared feature across the whole spectrum of mental disorders needs to be more thoroughly investigated. A study comparing 191 subjects with a schizophrenia spectrum disorder diagnosis and 81 patients with a current depressive episode (including 40 unipolar and 30 bipolar patients) found a clear group difference only for the subset of NSS pertaining to "movement disorders" and concluded that differences between disorders might be gradual rather than absolute (Boks et al. 2004). This concords with the study of Zhao et al., who found that schizophrenia patients scored higher than MDD patients regarding motor-coordination signs but did not significantly differ with regard to sensory integration and disinhibition (Zhao et al. 2013). In any psychiatric disorder, NSS are likely to reflect both trait and state characteristics of the disorder. However, the extent to which NSS represent either may differ between different mental disorders. In schizophrenia, NSS are in part determined by high genetic risk for the disorder (Picchioni et al. 2006). Neuroimaging studies have shown that increased NSS are associated with reductions in grey matter, as assessed via voxel-based morphometry, in particular concerning the prefrontal cortices and the cerebellum (Thomann et al. 2009; Mouchet-Mages et al. 2011). Likewise, increased NSS at 1-year follow-up in a sample of 20 patients with first-episode schizophrenia were associated with gray matter decreases of the sublobular claustrum, cingulate gyrus, cerebellum, frontal lobe and middle frontal gyrus (Kong et al. 2012). Importantly, a reduction in prefrontal gray matter is also a common finding in patients with MDD (Dai et al. 2019; Belleau et al. 2019). Thus, one may speculate that prefrontal structural aberrations may explain at least some of the neurological abnormalities found in the present study, but future fMRI studies will have to test this hypothesis. In our study, NSS were correlated with depressive symptoms independent of age. This is in agreement with a recent study that found a strong correlation of NSS with PTSD severity (Belrose et al. 2020), leading the authors to conclude that NSS might be biomarkers of severity. Accordingly, future research aimed at the identification of disorder-specific neurological abnormalities should ensure that different diagnostic groups are matched not only on the basis of socio-demographic factors but also with regard to illness severity. From a clinical standpoint, it is encouraging that we found NSS to remain unchanged after ECT, given that concerns regarding the safety of ECT persist in the literature (Read and Moncrieff 2022). Unfortunately, these concerns may discourage the clinical use of ECT even in cases when it is clearly indicated. Our data support the clinical safety of ECT from a neurological perspective and may reassure clinicians hesitant to apply ECT. Future research should probe whether NSS in MDD might be related to clinical and functional outcome, as has recently been demonstrated for schizophrenia (Ferruccio et al. 2021).

## Limitations

The comparison of NSS pre- and post-ECT may have been influenced by attrition bias. Moreover, we did not measure the exact interval between the last ECT session and the post-ECT NSS assessment, and raters were not blind to the subjects' diagnostic status. The explorative analyses, e.g., regarding potential associations of benzodiazepines/antipsychotics with NSS in the pre-ECT sample, were limited by small sample sizes in the compared groups. Finally, it should be noted that response and remission rates in the ECT group were quite low, due to our highly treatment-refractory sample in a tertiary referral center. Future studies should investigate potential associations of medication and NSS as well as potential ECT-induced NSS changes in subgroups of patients (e.g., ECT remitters or patients with catatonia, since catatonia usually responds rapidly to ECT (Luchini et al. 2015)) in larger samples.

## Conclusions

Patients with MDD showed significantly more neurological soft signs than healthy controls. These neurological abnormalities were found irrespective of illness duration and antidepressant treatment: NSS were increased in acutely depressed, unmedicated patients and chronically depressed, medicated patients. Investigating, for the first time, potential NSS changes after ECT, we found that the degree of soft neurological impairment did not change over the course of ECT. These results suggest that NSS may be trait rather than state markers of MDD and highlight the clinical safety of ECT.

## Supplementary Information

Below is the link to the electronic supplementary material.

Supplementary file2 (DOCX 54 KB)

## Data Availability

The data that support the findings of this study are available from the corresponding author upon reasonable request**.**
