# Supplementary material for: Neurological soft signs are increased in major depressive disorder irrespective of treatment
Source: J Neural Transm (Vienna). 2023 Feb 20;130(4):575–83. doi: 10.1007/s00702-023-02602-z (PMC10050027; doi:10.1007/s00702-023-02602-z)
Supplement: Supplementary file 1 — Supplementary file2 (DOCX 54 KB) [file 702_2023_2602_MOESM1_ESM.docx]

**Supplementary Material for Neurological Soft Signs Are Increased in Major Depressive Disorder Irrespective of Treatment**

by Rasmus Schülke^*^, Kyra Liepach, Anna Lena Brömstrup, Thorsten Folsche, Maximilian Deest, Stefan Bleich, Alexandra Neyazi, Helge Frieling, Hannah B. Maier

*Correspondence to: Rasmus Schülke, Department of Psychiatry, Social Psychiatry and Psychotherapy, Hannover Medical School, Carl-Neuberg-Str. 1, 30625 Hannover, Germany; schuelke.rasmus@mh-hannover.de

**Online Resource 1.**

**THE NEUROLOGICAL SOFT SIGNS (NSS) EXAMINATION** ^12–14^

Created by Tamara Gurvits, M.D.

Previously unpublished material, published with kind permission of the original authors.

**General Considerations**

Overview: The NSS examination derives from a classic neurological assessment that was extended to include tests of visuomotor and cognitive function. The choice of stimuli and emphasis was influenced by the methods and philosophy of the great Russian neuropsychologist, Alexander R. Luria. Unlike a traditional bedside neurological examination in which many parameters (such as gait or copying ability) are rated qualitatively, the NSS battery has explicit scoring criteria. All signs are scored from 0 (= normal) to 3 (= significantly impaired).

General Instructions for Examiners: After establishing rapport, explain that the battery will take approximately 60 minutes and that little or no physical discomfort will be involved. For the initial signs subjects will be required to remove their footwear and participate in stocking feet.

For each sign, the Examiner is provided with a brief description followed by instructions to read to the Subject verbatim. Needed equipment is specified. If a Subject shows any confusion or uncertainty, it is acceptable to repeat or paraphrase the instructions. Some signs require that the examiner first demonstrate the maneuver. In all cases it is acceptable for the Examiner to demonstrate on him/herself if the Subject is still having difficulty grasping what is required. Although the exact interactions may vary somewhat across subjects, the key point is that the NSS examination is a test of performance, and as such, the obtained score should not merely reflect variation in a given Subject's ability to comprehend what is required.

Scoring: Follow the explicit guidelines for each sign. Two independent scorers can rate the Subject's performance, and discrepancies can be resolved by consensus judgments.

Initial Instructions for Subjects: ”In a moment I will administer the Neurological Soft Signs battery. You will be given a series of tasks, puzzles and maneuvers to perform. Some will be timed. At the beginning of each sign, we will read some instructions explaining what you need to do. If you are unsure of what is expected, please don't hesitate to ask for clarification. The entire battery will take just about 60 minutes. Little or no physical discomfort is expected, but please let us know immediately if you become uncomfortable at any point for any reason. Ready to begin? Please try your best.”

**Protocol for administering and scoring the neurological soft signs examination.**

***For Signs #1-10, ask S to remove his/her shoes.***

**GAIT AND STATION**

| Sign # - Name | **#1 - Gait** |
| --- | --- |
| Description | S walks in normal fashion to the designated spot and then returns to starting point. |
| Equipment | Hallway, 3" masking tape. Lay a 15' long strip of tape about 2' from the closest wall. Lay a 12" strip of tape perpendicular to the long strip at each end as a starting and finish line. |
| Subject  Instructions | “Please walk along this hallway to the end of the tape and back in your normal manner.” |
| Scoring  Criteria | Look for signs of hemiplegia, spasticity, ataxia, steppage, waddling, limping, Parkinsonism, and wide-base gait. Watch for unsteadiness as S turns.  0 – normal gait  1 – mildly impaired  2 – moderately impaired  3 – severely impaired |
| Sign # - Name | **#2 - Walking on Tiptoe** |
| Description | S walks down the hallway and back on h/h tiptoes along the tape. |
| Equipment | Same as above. |
| Subject  Instructions | “Please walk on your toes down the hallway and back staying on the tape. Try not to put your foot down flat and try to stay on the line.” Demonstrate to S for a few steps. |
| Scoring  Criteria | Record an error each time S misses the line, puts h/h foot down flat, or requires support from the wall (touching/leaning):________  0 – no errors  1 – 1-2 errors  2 – 3 errors  3 – ≥ 4 errors |
| Sign # - Name | **#3 - Walking on Heels** |
| Description | S walks down the hallway and back on h/h heels along the tape. |
| Equipment | Same as above. |
| Subject  Instructions | “Please walk on your heels down and back on the tape. Try not to put your foot flat and try to stay on the line.” Demonstrate to S. |
| Scoring  Criteria | Record an error each time S misses the line, puts h/h foot down flat, or requires support from the wall (touching/leaning):________  0 – no errors  1 – 1-2 errors  2 – 3 errors  3 – ≥ 4 errors |
| Sign # - Name | **#4 -Tandem Gait** |
| Description | S walks down the hallway and back in heel-to-toe fashion along the tape. |
| Equipment | Same as above. |
| Subject  Instructions | “Please walk down and back on the tape placing each heel right in front of your toes with each step. Take your time. At the end of the tape please turn around and come back.” |
| Scoring  Criteria | Allow one misstep at the outset. Then record an error each time S misses the line or requires support from the wall (touching/ leaning):________  0 – no errors  1 – 1-2 errors  2 – 3 errors  3 – ≥ 4 errors |
| Sign # - Name | **#5 and #6 - Standing On One Foot (Right and Left)** |
| Description | S stands on one foot (first right, then left) without support for up to 20 seconds each. |
| Equipment | Stopwatch. |
| Subject  Instructions | “Please stand on your right/left foot only, until I tell you to stop.” |
| Scoring  Criteria | If after a slight disequilibrium at the start, S is able to gain balance for 20s, score as “1”.  0 – stable for 20s  1 – stable for 15-19s  2 – stable for 10-14s  3 – stable for <10s |
| Sign # - Name | **#7 and #8 - Romberg's Maneuver and Adventitious Overflow** |
| Description | S stands with eyes closed, feet together, arms outstretched held parallel to the floor, fingers spread apart for 30 seconds. |
| Equipment | Same as above. |
| Subject  Instructions | “Please stand with your eyes closed, feet together, arms outstretched parallel to the floor, and your fingers spread apart for as long as you can.” |
| Scoring  Criteria  #7 | 0 – stable for 30 seconds  1 – slight swaying  2 – marked swaying; subject steps to maintain balance  3 – unable to perform |
| Scoring  Criteria  #8 | 0 – absence of movements  1 – irregular fluttering movements of fingers only  2 – irregular fluttering movements extends to hand(s)  3 – involvement of entire upper extremity(ies) |

***For signs #9 - 54, S should be seated in a chair next to a desk.***

**MOTOR COORDINATION**

| Sign # - Name | **#9 and #10 - Foot Tapping (Right and Left)** |
| --- | --- |
| Description | S is seated with h/h feet resting comfortably, flat on the floor. S taps the ball of h/h foot as rapidly as possible while keeping the heel in place for 15 seconds. |
| Equipment | Chair w/armrest; Stopwatch. |
| Subject  Instructions | “Please sit down and rest your (right/left) foot flat on the floor. When I say "go", tap the ball of your foot as fast as you can on the floor while keeping your heel in place. I will tell you when to stop.” |
| Scoring  Criteria | Rate qualitatively for smoothness and speed of performance (e.g., absence of jerking, steady rhythm).  0 – normal performance  1 – mildly impaired  2 – moderately impaired  3 – severely impaired |

***Ask S to put h/h shoes back on.***

| Sign # - Name | **#11/12 and #13/14 - Finger Thumb Opposition (Right and Left)** |
| --- | --- |
| Description | S places both elbows on the armrest of a chair with fingers pointed upwards toward the ceiling. S touches the tip of each finger of one hand to the thumb in sequential order starting with the forefinger, continuing to the pinkie and then back to the forefinger. Continue for 15 repetitions. |
| Equipment | Chair w/armrest. |
| Subject  Instructions | “With your elbows on the armrest and your fingers of your right/left hand pointed towards the ceiling, please touch each finger to your thumb, starting with your forefinger, going to your pinkie and then back to your thumb. Repeat this sequence until I tell you to stop. There is no need to double touch the forefinger at the start of each new sequence.” |
| Scoring  Criteria  #11 | 0 – no disruption of motion, no mistakes  1 – slight disruption of motion or 1-2 mistakes  2 – moderate disruption of motion or 3 or more mistakes  3 – unable to perform |
| Scoring  Criteria  #12 | 0 – no observable movement in contralateral hand/fingers  1 – minor, inconsistent movements of contralateral hand/fingers  2 – minor but consistent contralateral movements  3 – consistent distinctive contralateral movements |
| Scoring  Criteria  #13 | 0 – no disruption of motion, no mistakes  1 – slight disruption of motion or 1-2 mistakes  2 – moderate disruption of motion or 3 or more mistakes  3 – unable to perform |
| Scoring  Criteria  #14 | 0 – no observable movement in contralateral hand/fingers  1 – minor, inconsistent movements of contralateral hand/fingers  2 – minor but consistent contralateral movements  3 – consistent distinctive contralateral movements |
| Sign # - Name | **#15 and #16 - Finger-Nose (Right and Left)** |
| Description | S fully extends right/left arm outward on the horizontal plane parallel to the floor and closes h/h eyes. S touches h/h index finger to the nose and returns to full extension. Repeat three times. Do not allow subject to support elbow on armrest or table. |
| Equipment | Same as above. |
| Subject  Instructions | “Please fully extend your right/left arm so that it is parallel to the floor. Now close your eyes, and touch the tip of your index finger to the tip of your nose three times, each time returning to full extension.” |
| Scoring  Criteria | Observe for dysmetria (side-to-side movement of the finger that increases in amplitude as the finger approaches the target), tremor and/or "past-pointing” to one side of the target.  0 – precise performance  1 – past point once and/or slight tremor/dysmetria  2 – past point twice and/or moderate tremor/dysmetria  3 – past point more than twice and/or severe tremor/dysmetria |
| Sign # - Name | **#17 and #18 - Nose-Pencil-Nose (Right and Left)** |
| Description | S fully extends right/left arm outward on the horizontal plane parallel to the floor with eyes open. S touches h/h index finger to the nose and returns to full extension. S then touches h/h index finger to the tip of the pencil and then returns to full extension. Repeat this sequence for three times. Do not allow subject to support elbow on armrest or table. |
| Equipment | Same as above; pencil. |
| Subject  Instructions | “Please fully extend your right/left arm so that it is parallel to the floor. Keep your eyes open. You are going to touch the tip of your index finger to the tip of your nose, return your arm to full extension, and then touch the tip of your finger to the tip of my pencil, and then back to the starting position. You will repeat this sequence this 3 times.” |
| Scoring  Criteria | Observe for dysmetria (side-to-side movement of the finger that increases in amplitude as the finger approaches the target), tremor and/or "past-pointing” to one side of the target.  0 – precise performance  1 – past point once and/or slight tremor/dysmetria  2 – past point twice and/or moderate tremor/dysmetria  3 – past point more than twice and/or severe tremor/dysmetria |
| Sign # - Name | **#19, #20, and #21 – Pronation/Supination (Right, Left, and Both)** |
| Description | S performs rapid, alternating movements with the palm and dorsum of h/h hand(s). Each hand is tested separately, then together, 10 times each. |
| Equipment | Chair w/armrest. |
| Subject  Instructions | “While you are sitting, please use your right/left/both hand(s) to touch your thigh alternately with your palm and the back of your hand, until I tell you to stop. Make sure to break contact with your thigh in between taps.” |
| Scoring  Criteria | 0 – no hesitation or mistakes in hand placement  1 – 1-2 hesitations or mistakes in hand placement  2 – 3-4 hesitations or mistakes in hand placement  3 – major disruption of motion |

**FRONTAL LOBES**

| Sign # - Name | **#23 and #24 - Fist/Palm/Side (Right and Left)** |
| --- | --- |
| Description | S touches h/h ipsilateral thigh first with the fist, then the palm, then the side of the hand using smooth, rhythmic motions. Make sure the S breaks contact with thigh between each change of hand motion. Repeat sequence 10x. |
| Equipment | Same as above. |
| Subject  Instructions | “Using your right/left hand, please touch your thigh or the table first with a fist, then your palm, then the side of your hand in a rhythmic motion. I will tell you when to stop.” Demonstrate 3x for each hand. |
| Scoring  Criteria | 0 – precise performance  1 – after first repetition, S hesitates no more than 2x in transition, and makes no more than 1 mistake in hand position  2 – after first repetition, S hesitates more than 2x times in transition, has difficulty in developing and maintaining a smooth, steady flow of movement, makes 2-4 errors in hand position, or has 3-4 total errors.  3 – major disruption of movements |
| Sign # - Name | **#27 and #28 - Palmomental Reflex (Right and Left)** |
| Description | S is asked to relax the facial musculature and open h/h mouth slightly. Stroke S's right/left palm at the thenar eminence with a key, and observe for the characteristic chin movement. Repeat 3x. |
| Equipment | Same as above; Key. |
| Subject  Instructions | “Keep your face relaxed, with mouth slightly open, and hold out your right/left hand, palm side up.” |
| Scoring  Criteria | 0 – no movement of chin muscles  1 – very slight movement  2 – distinct movement on one or two of attempts  3 – distinctive movement on all attempts |

**PARIETAL LOBES**

| Sign # - Name | **#29/31 and #30/32 - Astereognosis (Right and Left)** |
| --- | --- |
| Description | After S subject closes h/h eyes, press the objects below (in order) into h/h right/left outstretched palm, lightly compressing the fingers over the objects. Ask S to identify each object without palpating it. After both hands are tested, re-introduce any missed object, but now allow S to actively palpate the item. If no errors are made on #29, omit #31. If no errors are made on #30, omit #32. |
| Equipment | Chair with armrest.  Right Hand: Quarter Ring Key Button  Left Hand: Dime Watch Ring Screw |
| Subject  Instructions | 29/30: “Please close your eyes. I will place an object in your right/left palm. As I compress your fingers over the object try to identify it without moving your fingertips over it"  31/32: “This time it is OK to move your fingertips over the object. Try to identify it and take as much time as you need.” |
| Scoring  Criteria | #29: Circle Incorrect Responses: quarter, ring, key, button  If no errors are made on #29, omit #30.  #30: Circle Incorrect Responses: quarter, ring, key, button  0 – no errors  1 – 1 error  2 – 2 errors  3 – 3 or more errors  #31: Circle Incorrect Responses: dime, watch, ring, screw  If no errors are made on #31, omit #32.  #32: Circle Incorrect Responses: dime, watch, ring, screw  0 – no errors  1 – 1 error  2 – 2 errors  3 – 3 or more errors |
| Sign # - Name | **#33 and #34 - Graphesthesia (Right and Left)** |
| Description | S is seated with eyes closed. Examiner approaches S from behind and “writes” a number on the tip of h/h right/left outstretched index finger with the pencil/stylus. The numbers should be drawn in correct orientation to the S as he/she would read them.  Numbers for right hand: 1, 3, 7, 2, 8  Numbers for left hand: 7, 2, 1, 8, 3 |
| Equipment | Chair with armrest; Mechanical pencil or similar stylus. |
| Subject  Instructions | “Please close your eyes and hold out your right/left index finger. I will use a pencil tip to trace different numbers on the tip of your finger. After each one, tell me which number I have written". |
| Scoring  Criteria | #33: Circle Incorrect Responses: 1 3 7 2 8  #34: Circle Incorrect Responses: 7 2 1 8 3  0 – no errors  1 – 1 error  2 – 2 errors  3 – 3 or more errors |
| Sign # - Name | **# 35 - Extinction** |
| Description | Have S sit down and place hands palm down on his/her knees. S should have eyes closed while you touch the following places simultaneously: Right Face – Right Hand  Right Face – Left Hand  Right Hand – Left Hand  Left Face – Left Hand  Left Face – Right Hand  Left Face – Right Face |
| Equipment | Chair w/armrest. |
| Subject  Instructions | “Please sit down with your eyes closed and your palms down on your knees. In a moment I will touch you gently. After you feel my touch, please tell me what area or areas were touched.” |
| Scoring  Criteria | 0 – no errors  1 – 1 error  2 – 2 errors  3 – 3 or more errors |

**APRAXIA**

| Sign # - Name | **#44 - Reproduction of a house from matchsticks** |
| --- | --- |
| Description | Assemble a house out of matchsticks in front of subject while S is observing. Then, mix up the matches, and ask subject to recreate the house, while giving h/h more matches than needed. |
| Equipment | Same as above;12 Matchsticks |
| Subject  Instructions | “Please watch while I assemble a house from the matchsticks, and try to memorize the pattern. I will then mix up the house, and you will try and reassemble it. Take as much time as you need, and don’t pay attention to where the heads of the matches go.” |
| Scoring  Criteria | 0 – perfect reproduction  1 – slight distortion (one match misplaced or lost)  2 – moderate distortion (2 matches misplaced or lost)  3 – severe distortion or can’t perform task at all |

**TEMPORAL LOBES**

| Sign # - Name | **#52 – Tapping Rhythm Test** |
| --- | --- |
| Description | Have S close h/h eyes. Tap out the following fast and slow rhythms, have subject repeat them one by one. S can open eyes when tapping rhythms. 1. - -- --  2. --- --- ---  3. _ --- _ ---  4. ---- _ ---- _ ---- _ |
| Equipment | Chair w/armrest. |
| Subject  Instructions | “Please close your eyes and listen to the rhythm I am tapping. After each rhythm, please attempt to repeat it. You may open your eyes when repeating the rhythm.” |
| Scoring  Criteria | 0 – no errors  1 – 1 error  2 – 2 errors  3 – 3 or more errors |
| Sign # - Name | **#54 - Dysphasia** |
| Description | Have S repeat the tongue twister at a rapid rate. Allow up to three attempts to produce a correct response. If still incorrect, have S repeat it at a normal rate of speech. Any other tongue twister may be used as an alternative. |
| Equipment | Same as above. |
| Subject  Instructions | “Please repeat the following tongue twister quickly: 'Peter Piper picked a peck of pickled peppers'. |
| Scoring  Criteria | 0 – correct after first attempt  1 – correct after second attempt  2 – correct after third attempt  3 – unable to perform after several attempts |

***Hand S the NSS Stimulus/Response Booklet for the remaining signs***

**CONSTRUCTIONAL APRAXIA**

| Sign# - Name | **#39/40/41 - Drawing to Command: Clock/Daisy/House** |
| --- | --- |
| Description | Have S spontaneously draw each of three stimuli in its corresponding box on the response sheet. |
| Equipment | NSS Stimulus/Response booklet and pen. |
| Subject  Instructions | #39: “Please draw the face of a clock and include the numbers and hands.”  #40: “Please draw a daisy in a flowerpot.”  #41: “Please draw a house on perspective (three-dimensions).” |
| Scoring  Criteria | Features: Circular face; Numerals 1-12; Symmetrical # placement  0 – Three features  1 – Two features  2 – One feature only  3 – Unrecognizable/Gross distortion |
| Sign# - Name | **#50 - Geographical Agnosia** |
| Description | Present S with a map of the outline of Germany. Have S locate the listed cities on the map by inserting their corresponding number onto the page.  (Original version: Present S with a map of the outline of the USA. Have S locate the listed cities on the map by inserting their corresponding number onto the page.) |
| Equipment | Same as above. |
| Subject  Instructions | “Please find each listed city on the map and place its number on the map to mark its location.” |
| Scoring  Criteria | Circle incorrect Responses: Berlin München Köln Leipzig Hamburg Frankfurt a. M.  (Original version: Boston Miami Seattle San Francisco New Orleans Denver)  0 – no errors  1 – 1 error  2 – 2 errors  3 – 3 or more errors |
| Sign# - Name | **#53 - Drawing to Command: Face** |
| Description | Have S make a spontaneous drawing of the front view of a human face (i.e., not in profile). |
| Equipment | Same as above. |
| Subject  Instructions | “Please draw a human face.” |
| Scoring  Criteria | Circle Omitted Features: Eyes Pupils Eyebrows Nose Mouth Hair 0 – no omissions  1 – 1 omission  2 – 2 omissions  3 – 3 or more omissions |
| Sign# - Name | **#57/58 - Optic Agnosia- Page 1/2** |
| Description | Identical objects are embedded in each of six panels on each stimulus page. Have S view the panels and identify the objects. Page one is more difficult due to the higher density of the noise. |
| Equipment | Same as above. |
| Subject  Instructions | #57: “There are six panels on this page. There is a hidden object in each panel. Try to identify each object. Take your time. It is OK to move the page closer or farther to you.”  #58: “This page has the six same objects hidden. Identify as many as you can” (prompt for the items incorrectly identified on Page 1.) |
| Scoring  Criteria | #57: Circle Incorrect Responses: shovel tea kettle hammer key eyeglasses pitcher  0 – none incorrect  1 – 1 incorrect  2 – 2 incorrect  3 – 3 or more incorrect  If none incorrect on #57, score 3 on #58  #58: Circle Incorrect Responses: shovel tea kettle hammer key eyeglasses pitcher  0 – none incorrect  1 – 1 incorrect  2 – 2 incorrect  3 – 3 or more incorrect |
| Sign# - Name | **#59 - Money Road-Map Test** |
| Description | The examiner traces over the bold line and at each intersection the S states whether a L or R turn is required. |
| Equipment | Same as above. |
| Subject  Instructions | “This is a birds-eye view of a street map of an imaginary town. I will trace a path along the bold line. At each intersection, tell me whether a left or right turn is needed. Let's do some practice first at the lower right.” (If S makes an error on the practice trial provide the correct responses. Immediately following the practice trial begin the task proper). |
| Scoring  Criteria | # Incorrect Turns:  0 – no incorrect turns  1 – 1 or 2 incorrect turns  2 – 3 incorrect turns  3 – 4 or more incorrect turns |

**Online Resource 2.**

Neurological soft signs

| **Sign** | **p_adj_** | **HC vs. UDC** | **HC vs. ECT** | **UDC vs. ECT** |
| --- | --- | --- | --- | --- |
| Walking on heels | **0.001** | **0.004** | **0.000** | 1.000 |
| Graphesthesia (L) | **0.004** | 0.729 | **0.001** | **0.016** |
| Copy figure 2 | **0.007** | **0.000** | **0.024** | 0.292 |
| Foot tapping (R) | **0.011** | 0.396 | **0.001** | 0.094 |
| Finger-thumb Opposition (R) | **0.014** | 0.122 | **0.001** | 0.295 |
| Fist-palm-side (R) | **0.016** | **0.007** | **0.007** | 1.000 |
| Extinction | **0.016** |  |  |  |
| Drawing face | **0.020** | 1.000 | **0.005** | 0.066 |
| Pronation-supination (both) | **0.025** | **0.010** | 0.579 | 0.127 |
| Finger-to-finger (R) | 0.061 | 0.011 | 0.090 | 0.576 |
| Finger-to-finger (L) | 0.064 | 0.016 | 0.055 | 1.000 |
| Drawing flower | 0.064 | 0.066 | 0.022 | 1.000 |
| Tongue twister | 0.088 | 1.000 | 0.031 | 0.251 |
| Walking on tiptoe | 0.098 | 0.078 | 0.039 | 1.000 |
| Road map test of direction sense | 0.098 | 0.519 | 0.025 | 0.879 |
| Finger-thumb Opposition (L) | 0.127 | 0.354 | 0.034 | 1.000 |
| Fist-palm-side (L) | 0.135 | 0.166 | 0.071 | 1.000 |
| Palmomental reflex (R) | 0.135 | 0.063 | 0.048 | 1.000 |
| Finger-thumb Opposition (L), contralateral movement | 0.140 | 0.058 | 0.324 | 0.879 |
| Drawing house | 0.140 | 0.438 | 0.057 | 1.000 |
| Palmomental reflex (L) | 0.145 | 0.063 | 0.091 | 1.000 |
| Tandem gait | 0.145 | 0.125 | 0.068 | 1.000 |
| Copy figure 1 | 0.145 | 0.057 | 0.290 | 1.000 |
| Finger-nose (R) | 0.149 | 0.150 | 1.000 | 0.450 |
| Finger-thumb Opposition (R), contralateral movement | 0.160 | 0.122 | 0.105 | 1.000 |
| Romberg's maneuver | 0.173 | 0.137 | 1.000 | 0.387 |
| Foot tapping (L) | 0.202 | 0.300 | 0.185 | 1.000 |
| Astereognosis + (R) | 0.202 | 0.088 | 1.000 | 0.585 |
| Adventitious overflow | 0.211 | 0.242 | 1.000 | 0.417 |
| Gait | 0.232 | 0.150 | 0.579 | 1.000 |
| Astereognosis + (L) | 0.232 | 1.000 | 0.194 | 0.474 |
| Graphesthesia (R) | 0.232 | 0.312 | 0.282 | 1.000 |
| Astereognosis (L) | 0.299 | 1.000 | 0.288 | 0.486 |
| Copy figure 3 | 0.347 | 1.000 | 0.306 | 1.000 |
| Pronation-supination (R) | 0.419 | 0.717 | 1.000 | 0.540 |
| Drawing clock | 0.419 | 1.000 | 0.531 | 0.897 |
| Copy figure 6 | 0.419 | 0.558 | 1.000 | 0.633 |
| Copy figure 7 | 0.419 | 0.984 | 0.471 | 1.000 |
| Standing on one foot (R) | 0.419 | 0.627 | 0.420 | 1.000 |
| Optic agnosia | 0.458 | 1.000 | 0.570 | 0.882 |
| Optic agnosia + | 0.490 | 0.870 | 0.879 | 1.000 |
| Pronation-supination (L) | 0.575 | 0.684 | 1.000 | 1.000 |
| Copy figure 5 | 0.657 | 1.000 | 0.921 | 1.000 |
| House from matches | 0.657 | 1.000 | 1.000 | 1.000 |
| Astereognosis (R) | 0.672 | 0.936 | 1.000 | 1.000 |
| Finger-nose (L) | 0.751 | 1.000 | 1.000 | 1.000 |
| Geographic agnosia | 0.751 | 1.000 | 1.000 | 1.000 |
| Standing on one foot (L) | 0.829 | 1.000 | 1.000 | 1.000 |
| Tapping rhythm | 0.935 | 1.000 | 1.000 | 1.000 |
| Copy figure 4 | 0.955 | 1.000 | 1.000 | 1.000 |
| Online Resource 2. Between-group comparisons of individual neurological soft signs. Reported are FDR-adjusted p-values for Kruskal-Wallis tests and Bonferroni-corrected p-values for post-hoc pairwise Mann-Whitney U tests. Mann-Whitney U tests could not be performed for extinction since all HC and UDC subjects scored 0 (fields are left blank). HC: healthy controls, UDC: unmedicated MDD patients, ECT: medicated MDD patients scheduled for ECT. R: right, L: left. + indicates a more difficult version of the respective sign. | | | | |
